# Non-surgical interventions to control bleeding from arteriovenous fistulas and grafts inside and outside the hemodialysis unit: a scoping review

**DOI:** 10.1093/ckj/sfae089

**Published:** 2024-03-27

**Authors:** Elizabeth Milosevic, Adam Forster, Louise Moist, Faisal Rehman, Benjamin Thomson

**Affiliations:** Western University, Schulich School of Medicine and Dentistry, London, Ontario, Canada; Western University, Schulich School of Medicine and Dentistry, London, Ontario, Canada; Western University, Schulich School of Medicine and Dentistry, London, Ontario, Canada; Western University, Schulich School of Medicine and Dentistry, London, Ontario, Canada; Johns Hopkins University, Bloomberg School of Public Health, Baltimore, MD, USA

**Keywords:** arteriovenous fistulas, arteriovenous grafts, hemodialysis, hemorrhage, post-cannulation bleeding

## Abstract

**Background:**

Prolonged bleeding from arteriovenous fistulas (AVF) and arteriovenous grafts (AVG) associates with worse outcomes; Within the hemodialysis unit these outcomes include anemia and quality of life disruptions, and outside the hemodialysis unit includes fatal hemorrhage. However, various guidelines for AVF/AVG bleeding management inside and outside the hemodialysis unit lack consensus.

**Methods:**

A scoping review was conducted of four databases, from inception to 17 February 2024. The study population was hemodialysis patients experiencing bleeding from AVF or AVG. Studies that assessed non-operative management were included.

**Results:**

Sixteen studies met inclusion criteria. Most (14/16) addressed post-cannulation bleeding from AVF/AVG within the dialysis unit. Compared with standard dressings, hemostatic dressings (chitosan-, cellulose- or thrombin-based) decreased post-cannulation bleeding time at arterial and venous site 35.7%–84.0% (*P *< .05) and 38.5%–78.7% (*P *< .05), respectively. Use of chitosan-based dressings decreased percentage of patients bleeding 4-min post-cannulation by 16.3%–39.2%. One pilot observational study demonstrated no access thromboses or infections with short-term use of a compression device within the hemodialysis unit. However, the role of compression devices and tourniquets within the dialysis unit remains unclear, despite widespread use. Long-term AVF/AVG survival was not reported in any study. Limited research confirms that devices are effective in prevention of catastrophic out-of-hospital bleeding. It remains uncertain if device availability enhances patient confidence in managing out-of-hospital bleeding. This may impact patient choices around dialysis modality, access and transplant, but this remains uncertain.

**Conclusions:**

In hemodialysis patents with bleeding from AVF/AVG, several alternative dressings or devices decrease post-cannulation bleeding time within the hemodialysis unit. Existing research has not established criteria on when it might be appropriate to use specialized dressings. There is very limited research on methods to control bleeding from AVF/AVG outside the hemodialysis unit. More data are required before evidence-based guidelines can be made. Recommendations for future research are provided.

KEY LEARNING POINTS **What was known:**

- Prolonged bleeding from arteriovenous fistulas or grafts (AVF/AVG) in hemodialysis patients associates with worse clinical outcomes, whether inside or outside the hemodialysis unit.

**This study adds:**

- Hemostatic dressings (chitosan-, cellulose- or thrombin-based) consistently decrease post-cannulation bleeding time, compared with standard dressings, and may improve dialysis clearance at 1 year.
- While guidelines often discourage the use of tourniquets, they are commonly used in dialysis units, and may have physical properties that are preferred to the two-finger compression technique.
- Despite widespread advocacy for use, there is limited evidence to support use of devices to control AVF or AVG bleeding outside of hospital.

**Potential impact:**

- Directions of future research are proposed, including the evaluation of interventions on outcomes such as patient choices regarding transplantation, vascular access and dialysis modality.

## INTRODUCTION

Hemodialysis patients with an arteriovenous fistula (AVF) or arteriovenous graft (AVG) are at increased risk of bleeding due to the coagulopathy of end-stage kidney disease [1] and the use of systemic anticoagulation in the hemodialysis circuit [2]. The risk of prolonged bleeding specifically after needle removal from AVF or AVG cannulation sites is also impacted by needle position [2, 3] cannulation technique (buttonhole versus rope-ladder) [4], vascular access integrity, namely whether there is associated pseudoaneurysm/aneurysm, stenosis or infection [5]. Prolonged post-cannulation bleeding is associated with poor outcomes including increased rates of anemia [6], quality of life disruptions [7], and fatal catastrophic hemorrhage [8, 9]. Such bleeding also increases the likelihood of transfusion, which carries risks of alloimmunization and thus future organ rejection for patients awaiting kidney transplant [10]. For routine post-puncture bleeding, multiple guidelines assert that digital pressure should be applied after needle withdrawal [10–12] but there are limited and inconsistent recommendations about specific dressing types and when to use other hemostatic devices or techniques.

The risks of AVF/AVG bleeding outside of the hemodialysis unit have recently come to heightened attention from the UK “Put a Lid On It!” [13] and the Australia “Stop the Bleed” [14] campaigns. However, there remains limited evidence regarding management of bleeding from AVF/AVG outside the hemodialysis unit. When bleeding is more severe, or outside a dialysis unit, guidelines consistently indicate that vascular access hemorrhage is a surgical emergency [11, 15]. However, most guidelines do not provide in-depth recommendations regarding temporizing measures prior to operative management other than us of tourniquet and emergent assessment of the patient. Such measures are essential as catastrophic hemorrhage occurs most often outside the dialysis unit, in locations where immediate surgery is not possible [8, 9].

Consistent and evidence-based recommendations for the management of AVF/AVG bleeding both inside and outside the hemodialysis unit are needed, as bleeding outside of acute care environments may be potentially fatal [8, 9]. The aim of this review is to summarize the evidence regarding the use of non-surgical devices and techniques in the management of AVF/AVG bleeding, and to highlight opportunities for future research.

## MATERIALS AND METHODS

This review was informed by methodology proposed by the Joanna Briggs Institute [16] for scoping reviews, and adheres to the Preferred Reporting Items for Systematic Reviews and Meta-Analyses (PRISMA) extension for scoping reviews checklist.

### Search strategy and data sources

We searched the following databases (from inception to 17 February 2024): MEDLINE, Embase, Cochrane Central Register of Controlled Trials and Web of Science. Search strategy was not limited by language, year or study design. We also searched for published abstracts from the following international conferences: American Society of Nephrology (2003–23), Canadian Society of Nephrology (2012–23) and European Dialysis and Transplant Society (2010–23). Search terms included “arteriovenous graft,” “arteriovenous fistula,” “bleeding,” “hemorrhage,” “hemodialysis,” “dialysis” and “renal replacement therapy.” The Embase search strategy is included as Appendix 1.

Search results were imported into Covidence systematic review software (Veritas Health Innovation, Melbourne, VIC, Australia) for screening, review and data extraction.

### Study selection

Eligible studies included those that described non-surgical techniques for managing post-cannulation or spontaneous bleeding from AVF/AVG in HD patients. Studies were excluded if they focused on surgical management options, or on methods to prevent initial AVF/AVG bleeding. Studies that described bleeding from AVF/AVG that resulted from surgical procedures were also excluded. Finally, included studies were limited to those that focused on external bleeding as opposed to subcutaneous or intercompartmental bleeding, and those with full manuscripts available in English. Any patient location (inside or outside the dialysis unit) when post-cannulation bleeding occurred was considered.

Two reviewers (E.M. and B.T.) independently screened all titles and abstracts. The same individuals reviewed full-text articles to establish eligibility. Disagreements at all stages were resolved by discussion and consensus.

### Data extraction and synthesis

A data extraction form was developed. Study characteristics of interest included the type of study, country of publication, number of patients and intervention descriptions. Patient characteristics included age, sex and access type (AVF versus AVG). All outcomes were considered, but outcomes of special interest included those related to access (thrombosis, need for surgical intervention, access failure), anemia (need for blood transfusion, change in erythropoietin-stimulating agent or iron dose), quality of life, changes in dialysis modality or kidney transplantation decision, hospitalization and death.

Case reports that described an intervention in only one patient were also included.

### Study quality

The quality of non-randomized studies was evaluated using the ROBINS-E assessment tool [17]. The quality of randomized studies was evaluated using the Jadad score [18].

### Ethics

The project was exempt for the requirement to seek research ethics board review as it fell within Tri-Council Policy Statement (TCPS)-2 article 2.4: “REB review is not required for research that relies exclusively on secondary use of anonymous information, or anonymous human biological materials, so long as the process of data linkage or recording or dissemination of results does not generate identifiable information.”

## RESULTS

### Study selection

**Figure 1:**
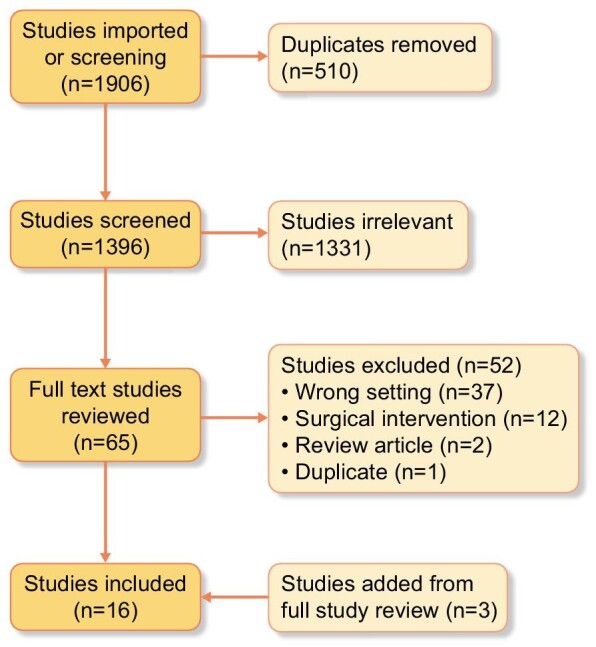
PRISMA chart for scoping review.

Search of MEDLINE, Embase, Cochrane, Web of Science and conference abstracts yielded 1906 studies (Fig. 1). There were 510 duplicates removed, leaving 1396 studies. Title and abstract review identified 1331 irrelevant references, yielding 65 studies for full-text review. There were 52 studies excluded for wrong setting (*n* = 37), for describing surgical interventions (*n* = 12), for being review articles (*n* = 2) and for being duplicate (*n* = 1). During full-text review, three additional studies were identified for inclusion, by review of publication reference lists. This yielded 16 studies for inclusion.

### Study characteristics

Most (14/16) studies evaluated bleeding within the hemodialysis unit (Table 1). Both studies outside the hemodialysis unit assessed management of potentially catastrophic AVF or AVG bleeding using mechanical devices.

**Table 1:** Study characteristics.

Characteristic	Number of studies (%)
Bleeding location	
Inside hemodialysis unit	14 (88)
Outside hemodialysis unit	2 (12)
Design	
Observational	8 (50)
Case reports	3 (19)
Case series	2 (13)
Retrospective observational	2 (13)
Prospective observational	1 (6)
Interventional	8 (50)
Prospective crossover	6 (38)
Prospective randomized	2 (13)
Type	
Manuscript	13 (81)
Conference Abstract	3 (19)
Number of centers	
Single center	13 (81)
Multicenter	3 (19)
Country	
USA	8 (50)
France	2 (13)
Israel	2 (13)
Spain	1 (6)
Iran	1 (6)
Japan	1 (6)
Nigeria	1 (6)
Hemostatic device/technique	
Specialized dressings	
Chitosan based	3 (19)
Cellulose based	2 (13)
Thrombin soaked	1 (6)
TXA impregnated	1 (6)
Poly-N-Acetyl glucosamine based	1 (6)
Multilayer non-impregnated	1 (6)
Tourniquet	3 (19)
Clamp	2 (13)
Bottle cap	1 (6)
Multiple methods	1 (6)
Vascular access type	
AVF only	7 (44)
AVF and AVG	7 (44)
Not stated AVF or AVG	2 (13)

There were observational studies (*n* = 8) and interventional trials (*n* = 8) included (Tables 1 and 2). Interventional trials included prospective crossover (*n* = 6) and prospective randomized (*n* = 2) trials. Observational studies included case reports (*n* = 3), case series (*n* = 2), retrospective observational (*n* = 2) and a prospective observational study (*n* = 1). Most took place in the USA (8/16), Europe (3/16) or Western Asia (3/16). Hemodialysis vascular access type was exclusively AVF (7/16), mixed AVF and AVG (7/16), or not stated (2/16). Chitosan-based specialized dressings and tourniquets each had three studies describing their use. The majority of studies evaluated specialized dressings (9/16) while the remaining evaluated mechanical devices such as tourniquets (3/16), clamps (2/16) or bottle cap (1/16).

**Table 2:** Studies of hemostatic dressings or devices to control post-cannulation bleeding from AVF or AVG.

Study (country)	Study design	Intervention/observation	Patients (*n*)	HD sessions/patient	Age (mean, range)	Sex (% female)	AVF:AVG	Results	Risk of bias
Inside hemodialysis unit									
Specialized dressings: chitosan-based									
Bachtell *et al*., 2006 (USA)	SC PCS (M)	Chitosan-based dressing vs conventional bandage	50	2	NR, 24–85	48.0	68:32	Hemostasis at 4 min post-removal of needles achieved more frequently (86% vs 72%, *P *= .040). Compression straps used less often (14% vs 28%, *P* = .052)	Serious
Suzuki *et al*., 2013 (Japan)	SC PRT (CA)	Chitosan-based dressing vs conventional bandage	30	3	NR, NR	40.0	100:0	Hemostasis at 4 min post-removal of needles achieved more frequently (100% vs 60.8%, *P *< .01)	No info
Misgav *et al*., 2017 (Israel)	SC PCS (M)	Chitosan-based vs alginate dressing	15	10	76.7, 54–93	33.3	60:40	Bleeding time reduced at AS (3.00 vs 18.76 min, *P *< .001) and VS (2.83 vs 13.28 min, *P *< .001)	Critical
Specialized dressings: cellulose-based									
Bizari *et al*., 2019 (Iran)	SC PCS (M)	Cellulose-based sponge vs conventional gauze	60	2	55.2, NR	41.7	100:0	Bleeding time reduced at AS (3.15 vs 12.74 min, *P *< .001) and VS (2.86 vs 10.54 min, *P *< .001)	Moderate
Kliuk-Ben Bassat *et al*., 2021 (Israel)	SC PRT (M)	Cellulose-based gauze vs conventional gauze	49	1 year^a^	65.2, NR	29.2	100:0	Outcome 1: bleeding time reduced by 6.38 min at AS (*P *< .001) and 3.99 min at VS (*P *< .001). Outcome 2: dialysis adequacy increased (1.73 vs 1.53, *P *= .047)	Jadad 2/5
Specialized dressings: other types									
Boulanger *et al*., 2014 (France)	MC PCS (M)	Multilayer non-impregnated bandage (Iris) vs conventional gauze	64	9	68, 22–88	44.0	98.4:1.6	Persistent bleeding at 3 min reduced at AS (18% vs 54% at Week 1, *P *< .05; 18% vs 56% at Week 3, *P *< .05) and VS (23% vs 45% at Week 1, *P *< .05; 23% vs 45% at Week 3, *P *< .05)	Moderate
Vaziri, 1979 (USA)	SC PCS (M)	Thrombin-soaked gauze vs diluent-soaked gauze vs conventional gauze	12	9	NR, 42–70	33.3	50:50	Mean bleeding time 3.2 vs 7.0 vs 6.4 min (*P *< .001 for thrombin-soaked vs either other dressing)	Moderate
Ghaffari *et al*., 2010 (USA)	SC PCS (CA)	Poly-N-acetyl glucosamine–based patch vs conventional gauze	30	2	NR, NR	NR	NR	Mean hemostasis time reduced at AS (6.3 vs 9.8 min, *P *= .01) and VS (5.6 vs 9.1 min, *P *= .01). Mean hemostasis time at AS and VS decreased in patients on anticoagulation (*P *< .05) but not antiplatelets (*P *> .05)	No info
Eberle *et al*., 2020 (USA)	SC Obs (M)	Use of transexamic acid impregnated gauze	1	1	84, NR	0.0	100:0	Bleeding stopped within 30 minutes of applying tranxexamic acid impregnated gauze, in patient whose AVF bled 30 min post-dialysis	No info
Devices or techniques									
Lala *et al*., 1985 (Nigeria)	SC Obs (M)	Describe management of torrential bleeding in one patient (tourniquet)	1	NR	NR, NR	100.0	100:0	Severe bleeding during AVF venepuncture led to attempted local pressure, IV vitamin K and underrunning suture—all were unsuccessful. Tourniquet applied and successfully stopped bleeding before patient transfer to operating room	No info
Inui *et al*., 2017 (USA)	MC Obs (M)	Describe management of patients presenting to hospital with hemodialysis access bleeding	26	NR	NR, NR	54.0	30.8:69.2	Hemodialysis patients with prolonged, high volume AVF or AVG bleeding underwent sutures (*n* = 14) or tourniquet (*n* = 4) with successful bleeding control in all cases	Serious
Sallee *et al*., 2021 (France)	MC Obs (M)	Describe cannulation and hemostasis practices and knowledge	3588	NR	70.0, 18–97	39.0	100:0	There were at least 10 different dressings used to control AVF or AVG bleeding. Mechanical compression devices commonly used with short (<10 min) and long (>10 min) bleeding (12.7 and 13.2%, respectively)	No info
Cristobal *et al*., 2021 (Spain)	SC Obs (M)	Adjustable clamp vs standard 2-finger pressure on access	15	1–10 per patient, total 51	71.3, NR	60.0	86.7:13.3	Pressure between venous and arterial cannulation sites lower with adjustable clamp than 2-finger manual pressure (*P *< .001). Manual pressure shows pressure gradient along access, while clamp pressure more consistent	Serious
Abidian *et al*., 2023	SC Obs (CA)	Compression device placed on AVF/AVG	28	166 in 28 patients	NR, NR	NR	NR	No access infections or thrombosis noted. Only 1 episode (0.03% times used) post-cannulation bleeding >15 min. Patient satisfaction high (4.6/5 on Likert scale). Patients highly preferred over manual compression	No info
Outside hemodialysis unit									
Zietlow *et al*., 2015 (USA)	SC Obs (M)	Describes use of tourniquets for bleeding control by rural medical transport service	9	NR	NR, 1–93	22.0	NR	7% (*n* = 9) of all patients requiring tourniquet application had AVF or AVG. Tourniquets were 98.7% successful and hemostatic gauze 95% successful at controlling all-cause bleeding	No info
Greenstein *et al*., 2023 (USA)	SC Obs (M)	Describes use of bottle cap to control severe bleeding from AVF	1	NR	71, NR	100.0	100:0	Large AVF bleeding outside of hospital led to bottle cap placement over AVF. This stopped bleeding. Upon removal of bottle cap, bleeding returned during surgical AVF repair	No info

AS, arterial site of AVF or AVG; AVF, arteriovenous fistula; AVG, arteriovenous graft; CA, conference abstract; HD, hemodialysis; M, manuscript; MC, multiple center; NR, not reported; Obs, observational; PCS, prospective crossover study; PRT, prospective randomized trial; SC, single center; VS, venous site of AVF or AVG.

### Specialized dressings

Most studies that evaluated the use of specialized dressings (8/9) were prospective and most (8/9) were single center. The number of study patients ranged from 1 to 60. Follow-up time varied from 1 to 10 hemodialysis treatments. Age was inconsistently reported, with only five reporting mean age and four reporting age range. All studies reporting patient sex (8/8) had a majority male population. All studies, with the exception of one that did not report on access type, included patient populations exclusively with AVF (4/9) or with a mixture of AVF and AVG (4/9).

Post-cannulation bleeding time was compared between standard and specialized dressings in four studies; use of specialized dressings decreased bleeding time at arterial site by 35.7%–84.0% (9.6–6.3 min, 12.7–3.2 min, 18.8–3.0 min, *P *< .05 for each comparison), at venous site by 38.4%–78.7% (9.1–5.6 min, 10.5–2.9 min, 13.2–2.8 min, *P *< .05 for each comparison) and at unspecified site by 53.1% (6.4–3.0 min, *P *< .05). These four studies included one with chitosan-based dressings, one with cellulose-based gauze, one with thrombin-soaked gauze and one with Poly-N-acetyl glucosamine–based patch. The percentage of patients with bleeding 4-min post-cannulation was assessed in two studies; use of specialized chitosan-based dressings decreased the percentage of patients bleeding at 4 min by 16.3%–39.2% (*P *< .05 for each of two studies). In a 3-week prospective study, patients underwent conventional manual compression for Weeks 1 and 3, and IRIS bandage compression in Week 2; persistent bleeding 3-min post-cannulation was lower in Week 2 (IRIS) than Weeks 1 and 3 (standard bandage) at the arterial sites (18% vs 54% and 56%, *P *< .05 for both comparisons) and venous sites (23% vs 45% and 45%, *P *< .05 for both comparisons).

Dialysis clearance improved in 1 study after 1 year of specialized cellulose-based bandage use (sKt/V 1.76 vs 1.53, *P *= .047), without change in dialysis duration or filter type. This was suggested to be due to either improved preservation of vascular access or sample error.

No adverse effects of using specialized dressings were reported. No study assessed cost-effectiveness of specialized dressings’ use. No study established criteria on when it might be appropriate to use specialized dressings.

### Mechanical devices

All studies that described the use of mechanical devices to manage post-cannulation bleeding were observational (7/7).

The use of tourniquets (3/7) was the most frequently reported. One study described their use outside the hemodialysis unit, in a rural medical transport service in the USA [19]. This case series of patients managed by a rural medical transport service demonstrated that of all patients needing tourniquet administration, 7% (*n* = 9) were due to bleeding from AVF or AVG. Tourniquets were successful for 98.7% of all cases of bleeding in this study, but it was not stated whether the unsuccessful cases were with AVF or AVG. The second study with tourniquet use was in a patient in Nigeria with catastrophic bleeding after AVF venepuncture [20]. In this patient, AVF venepuncture precipitated severe bleeding. Local pressure, intravenous vitamin K and suturing of the AVF were unsuccessful at stopping bleeding, and ultimately a tourniquet was applied until the patient reached the operating room for surgical AVF closure. The third study with tourniquet use was in hemodialysis patients with prolonged, high volume AVF or AVG bleeding [21]. This was a case series of patients with either AVF or AVG, with prolonged post-cannulation bleeding, who underwent either tourniquet (*n* = 4) or suture (*n* = 14) placement. Bleeding was stopped in all cases. There were no long-term outcomes reported, such as long-term fistula patency or need for surgical intervention.

One study [22] of 15 patients compared the use of an adjustable clamp to standard two-finger pressure to stop post-cannulation bleeding. The pressure at the vascular access site was quantified during the maneuvers. Both maneuvers successfully stopped bleeding, but the clamp had a lower pressure requirement, and a more consistent pressure across the entire access.

One study [23] assessed the use of a compression device, placed on AVF or AVG post-cannulation. No access infections or thromboses were reported after 166 dialysis treatments in 28 patients. Only one dialysis treatment (0.03%) using the device had bleeding longer than 15 min. Patient satisfaction was high (4.6/5 on Likert scale). However, there was neither a control group nor a historical comparison in this study.

One study [24] evaluated practices of hemodialysis nurses in France in responding to post-cannulation bleeding for over 3000 patients. Nurses reported using at least 10 different dressing types, and mechanical compression devices (bracelets, clips or tourniquets) were commonly used for both short and long bleeding times (12.7% and 13.2%, respectively).

One case report [25] used a bottle cap to control severe bleeding in a hemodialysis patient whose AVF started bleeding outside of hospital. Application of the bottle cap successfully stopped the bleeding, until it was removed in the operating room to surgically repair the AVF.

No adverse effects of using mechanical devices were reported.

### Study quality

There were two randomized trials, but only one had sufficient information to be graded, and had low Jadad grade (2/5). Of the 14 non-randomized studies, 6 had insufficient information to be graded. The remainder were at moderate (*n* = 3), serious (*n* = 3) or critical (*n* = 1) risk of bias (Table 2).

## DISCUSSION

**Figure 2:**
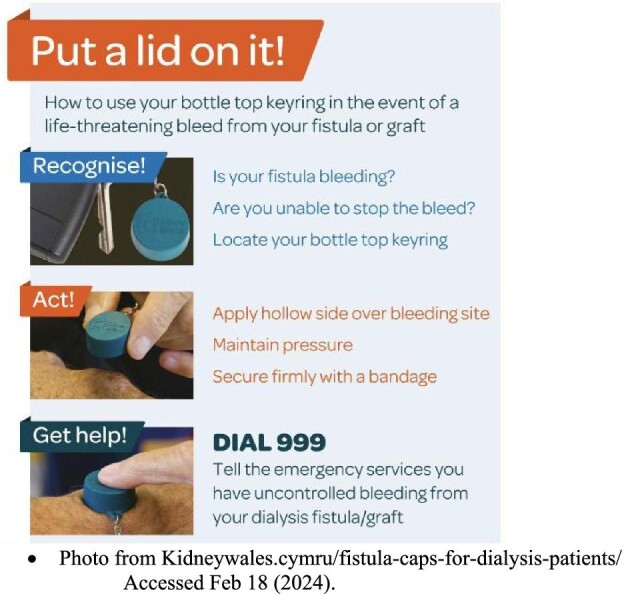
Instruction sheet for “Put a Lid On It!” campaign. Photo from Kidneywales.cymru/fistula-caps-for-dialysis-patients (accessed 18 February 2024).

This review confirms that the use of specialized dressings within the hemodialysis unit reduces bleeding time, compared with standard dressings. However, this review found minimal study of the management of AVF/AVG outside of a hospital setting [19, 25] despite significant recent attention paid to this topic from the UK “Put a Lid On It!” [13] and the Australian “Stop the Bleed” campaigns [14] (Fig. 2). Existing guidelines are inconsistent regarding the use of compression devices [10–12, 26, 27], but this review suggests they may have a role in managing severe bleeding outside a hospital setting. Compression devices may also have a role within the hemodialysis unit given widespread use [24], and advantageous physical properties [22]; this is an important finding given existing guidelines are inconsistent regarding the use of compression devices [10–12, 26, 27].

Factors that increase the likelihood of AVF or AVG bleeding are well described. Cannulation technique is important [28], with the use of blunt needles [29] and bevel down positioning [3, 30] carrying less risk. AVG are also more likely to bleed than AVF [31, 32], with the type of graft material also potentially having an impact [33]. The use of buttonhole needling technique may increase bleeding rates [34, 35], although this finding is not consistent [36]. Medications such as antiplatelets or anticoagulants also increase risk [5]. While there is relatively extensive literature on how to prevent AVF/AVG bleeding, the lack of research on how to manage bleeding when it happens remains a persistent gap limiting evidence-based care in hemodialysis patients.

This study identified multiple types of specialized dressings, most notably those derived from cellulose and chitosan, that reduce post-cannulation bleeding in hemodialysis patients. Given increased costs, these dressings must be reserved for select circumstances. However, the current vascular access guidelines do not provide specific recommendations about which dressing types to use and when to use them, aside from occasionally mentioning that hemostatic dressings can be considered in instances of prolonged post-cannulation bleeding. This review confirms that there is a reduction in bleeding time with use of specialized, as opposed to standard dressings. However, whether these reductions in bleeding time are clinically meaningful is uncertain, since there are no data regarding long-term access patency or need for access intervention. Furthermore, there is only one study that has suggested prolonged AVF/AVG bleeding time may associate with decreased dialysis adequacy; authors in that study admitted that this finding could be a sample error. As such, there can be no evidence-based recommendations on when to appropriately use the specialized dressings.

Given the paucity of evidence to support the use of specialized hemostatic dressings, there is significant variability in the dressings used in hemodialysis units, with French nurses reporting the use of over 10 different dressings used in one study [24]. More research is thus needed to compare these specialized dressings and determine their appropriate indications for use.

Studies that evaluated the use of devices to manage access bleeding were conducted predominantly outside hemodialysis unit settings. Most of the bleeding in these cases was severe, in which case maintenance of fistula patency is considered secondary to prevention of fatal exsanguination. While tourniquets are effective in this context as a temporizing measure, it remains unknown if clamps or tourniquets could be useful in managing post-cannulation bleeding within hemodialysis units. A survey of nurses showed that tourniquets are indeed used in hemodialysis units [24], despite best practice guidelines definitively recommending against them [12, 26, 27]. The conventional rationale for avoiding clamp and tourniquet use has centered around the risk of access thromboses or failure, but there is good reason to reevaluate this. Thrombosis formation is more probable when blood flow is turbulent rather than laminar [37]. Passage of blood through a fistula should thus have as consistent a pressure gradient as possible to minimize blood flow turbulence. The use of adjustable clamps on vascular access sites associated in one study with more consistent pressure measurement across the fistula, compared with two-finger compression technique [22]. Similarly, chronic use of a sleeve compression device on aneurysmal parts of AVF has not been found to be associated with aneurysmal expansion, increased bleeding or thrombosis [38]. It is thus possible that clamp use to manage post-cannulation bleeding could lead to lower AVF thrombosis rates. This should be prospectively evaluated.

The lack of research on management of post-cannulation bleeding may reflect the perception that severe AVF or AVG bleeding events are rare and simply mandate surgical intervention. Reported rates range from 0.5 to 1.0 fatal access bleeds per 1000 patient dialysis-years [9, 39]. It is unclear what proportion of these events are suicides [40, 41], but it is possible that modification of program policies and education to equip patients and caregivers with tourniquets or other devices might prevent some of these fatalities [39, 42]. Rural emergency workers who applied tourniquets to bleeding AVF or AVG were successful at stopping bleeding almost 100% of the time. While it is unknown whether these patients ultimately required surgical intervention, the tourniquet was effective to prevent fatal bleeding outside the controlled hemodialysis unit setting [19]. It is also essential to recognize that fatal bleeding is not the only undesirable outcome related to hemodialysis vascular access bleeding. Hemodialysis patients have high levels of concern regarding the sight of blood from their vascular access [43], and it likely has an impact on choosing whether to do dialysis at home or in a supervised setting [44]. However, it remains unknown whether the availability of post-cannulation bleeding devices modifies patient dialysis modality choice, and specifically whether it increases uptake of home dialysis modalities. Given the importance of increasing home dialysis rates, and reducing the proportion of hemodialysis patients who use central venous catheters for definitive vascular access, it is critical that devices that are used to minimize bleeding are studied to establish any effect on these important outcomes. Similarly, whether the education about such a device in the pre-dialysis chronic kidney disease clinic modifies patient choices about dialysis modality or vascular access remains unknown.

**Figure 3:**
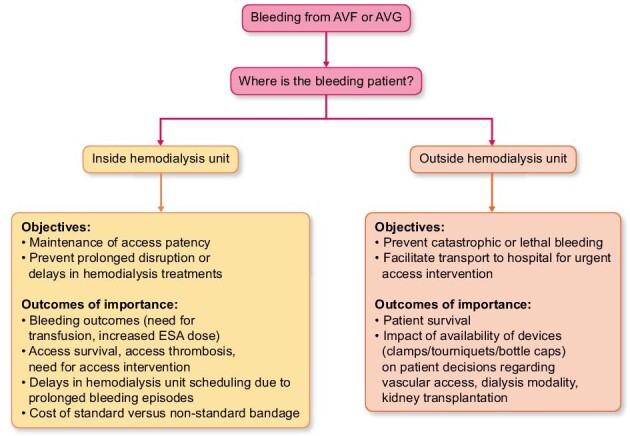
Management of post-cannulation bleeding: proposed research direction.

This study reinforces the paucity of data informing current guidelines on management of post-cannulation bleeding. Despite a comprehensive search of the published medical literature using multiple databases, only 15 relevant publications were identified, and these varied greatly in the methods used to control bleeding and general methodology. The study highlights both a critical need for additional research about post-cannulation bleeding and the importance of consensus development to better support and guide clinicians (Fig. 3). Future research should focus on the impact of clamps on AVF thrombosis and long-term patency in hemodialysis, along with the impact of hemostatic devices on patient quality of life and decision-making regarding dialysis modality and vascular access. We acknowledge a likely reporting bias, as published cases are more likely to reflect management successes rather than failures. Given the widespread use of devices in the hemodialysis unit [24], it is likely that both successes and failures are underreported.

In summary, this review highlights the need for further study of devices and techniques to manage post-cannulation bleeding inside and outside the hemodialysis unit, with clinical outcomes expanded to include not only fatal hemorrhage, but also patient-centered outcomes such as choices regarding vascular access and dialysis modality.

## Data Availability

The data underlying this article are available in the article.
